# Death of Mr. Tyrrell

**Published:** 1843-06-24

**Authors:** 


Death of Mr. Tyrrell.—We have to announce, with regret, the death of Mr. Tyrrell, of St. Thomas's Hospital, which took place suddenly on Tuesday last. Mr. Tyrrell, we believe, had for a long time laboured under symptoms of an affection of the heart.

